# Impact of implementing Dutch versus European guideline risk factor targets in older patients with ischaemic heart disease

**DOI:** 10.1007/s12471-023-01823-x

**Published:** 2023-10-23

**Authors:** Tinka J. van Trier, Marjolein Snaterse, Ron M. C. Herings, Jetty A. Overbeek, Ron J. G. Peters, Harald T. Jørstad

**Affiliations:** 1 Department of Cardiology, Amsterdam University Medical Centres, location Academic Medical Centre, Amsterdam, The Netherlands; 2 Department of Epidemiology and Data Science, Amsterdam Public Health research institute, Amsterdam University Medical Centres, location Free University Medical Centre, Amsterdam, The Netherlands; 3 PHARMO Institute for Drug Outcomes Research, Utrecht, The Netherlands; 4 Department of General Practice, Amsterdam University Medical Centres, location Free University Medical Centre, Amsterdam, The Netherlands

**Keywords:** Secondary prevention, Elderly, Ischaemic heart disease, Blood pressure, Cholesterol, Risk factor management

## Abstract

**Background:**

In patients with ischaemic heart disease (IHD) aged > 70 years, Dutch and European guidelines recommend different treatment targets: low-density lipoprotein cholesterol (LDL-c) < 2.6 versus < 1.4 mmol/l and systolic blood pressure (SBP) < 140 versus < 130 mm Hg, respectively. How this impacts cardiovascular event-free life expectancy has not been investigated. The study objective was to compare estimated lifelong treatment benefits of implementing Dutch and European LDL‑c and SBP targets.

**Methods:**

Data from patients aged 71–80 years hospitalised for IHD in 2017–2019 were extracted from the PHARMO Database Network, which links primary and secondary healthcare settings, with follow-up until 31 December 2020. Potential benefit according to treatment strategy (in gain in event-free years) was estimated using the SMART-REACH model.

***Results*:**

Of the 3003 eligible patients, 1186 (39%) had missing LDL‑c and/or SBP measurements. Of the 1817 included patients (36% women, median age at event: 74 years (interquartile range (IQR): 72–77), 84% achieved the Dutch targets for both LDL‑c and SBP; for European targets, this was 23% and 61%, respectively. If Dutch targets were met for LDL‑c and SBP (*n* = 1281), the additional effect of reaching European targets was a median gain of 0.6 event-free life years (IQR: 0.3–1.0). The greatest effect could be reached in patients not reaching Dutch targets (*n* = 501), with a median gain of 0.6 (IQR: 0.2–1.2) and 1.7 (IQR: 1.2–2.5) event-free years with Dutch versus European targets.

***Conclusion*:**

In patients aged > 70 years with IHD, implementation of European targets resulted in a greater gain of event-free years compared with Dutch targets, especially in patients with poorer risk factor control. The considerable number of patients with missing risk factor documentation suggested additional opportunities for risk reduction.

**Supplementary Information:**

The online version of this article (10.1007/s12471-023-01823-x) contains supplementary material, which is available to authorized users.

## What’s new?

- After hospitalisation for ischaemic heart disease, a considerable proportion (39%) of patients aged 71–80 years had no low-density lipoprotein cholesterol (LDL-c) and/or systolic blood pressure (SBP) measurements.
- Dutch targets for LDL‑c (< 2.6 mmol/l) and SBP (< 140 mm Hg) were both met by 84% of the study population, while 23% met the European target for LDL‑c (< 1.4 mmol/l) and 61% met that for SBP (< 130 mm Hg).
- The greatest treatment effect—i.e. a gain of median 1.7 event-free years—could be attained by implementing European LDL‑c and SBP targets, with higher potential gains in patients with poorer risk factor control.

## Introduction

Patients with ischaemic heart disease (IHD) are at high risk of subsequent events or death, particularly when at higher age and with elevated risk factors [1]. Effective modification of cardiovascular disease (CVD) risk factors—e.g. lowering the level of low-density lipoprotein cholesterol (LDL-c) and systolic blood pressure (SBP)—reduces this risk [2, 3]. To assist clinicians in optimal risk factor management, both national (Dutch) and international (European) guidelines recommend treatment targets for these risk factors. However, there is disparity between the national and international guideline treatment targets. For patients aged > 70 years, the 2019 European Atherosclerosis Society (EAS) and 2021 European Society of Cardiology (ESC) prevention guidelines recommend an ultimate LDL‑c target of < 1.4 mmol/l and an SBP target of < 130 mm Hg (level 1A) [4, 5]. In contrast, Dutch guidelines recommend more lenient treatment targets in these patients, i.e. LDL-c < 2.6 mmol/l and SBP < 140 mm Hg (if tolerated) [6].

While reaching stricter LDL‑c and SBP targets is generally thought to effectively and safely reduce residual risk of recurrent CV events in the elderly [7, 8], it can also increase the risk of side effects, next to being time-consuming and potentially more costly. Especially in patients at older age, who are generally at high CVD risk and have limited life expectancy, estimating benefit in terms of gain in healthy years may be more relevant to guide treatment choices compared with risk reduction quantifications. Therefore, when considering which guideline target to adhere to in clinical practice, insights into the potential benefit of implementing the Dutch versus European guidelines in real-world patients may assist clinical decisions.

**Fig. 1:**
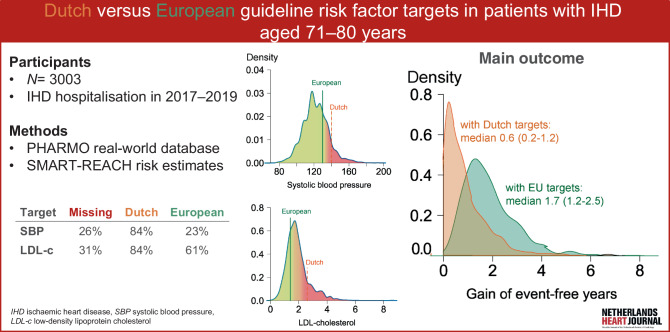
Infographic

In the current study, we investigated the impact of implementing the Dutch and European LDL‑c and SBP targets and compared estimated lifelong treatment benefits of both strategies in a large, real-world population study (Fig. 1).

## Methods

### Source population

We used data from the PHARMO Database Network, a population-based network of electronic healthcare databases that combines ongoing anonymous data collection from different primary and secondary healthcare settings in the Netherlands. The longitudinal nature of the PHARMO Database Network enables follow-up of 7 million patient journeys through different healthcare settings, via linkage of national databases and registries. Study populations are created from the Database Network using the data sources needed to address specific study objectives. In general, the population included in the PHARMO Database Network is representative of the Dutch population with respect to age and gender, but the database contains a relatively large proportion of individuals > 80 years, possibly due to an increased use of healthcare in older age [9].

### Study population and data extraction

For the current analysis, healthcare settings included in the data extraction file from the PHARMO Database Network were general practices, clinical laboratories and hospitals. A retrospective cohort was extracted that included patients aged 71–80 years who were hospitalised for IHD in 2017, 2018 or 2019. For the primary analysis, we excluded patients with missing LDL‑c and/or SBP measurements. The use of the study-specific dataset from the PHARMO Database Network was controlled by an independent privacy and governance board, the Compliance Committee [9].

We analysed data as of 1 January 2017, with individual index dates defined as the first hospitalisation with a primary diagnosis of IHD. Follow-up was until death or end of study (31 December 2020). We extracted individual-level data from the general practitioner’s notes on the patient’s age, sex, International Classification of Primary Care (ICPC) codes for medical history, smoking status, body mass index (BMI) and blood pressure measurements. Furthermore, we collected information on recurrent hospitalisations and outpatient clinic visits with corresponding primary diagnosis and laboratory results. Medical history was defined using ICPC codes and/or International Classification of Diseases (ICD)–10 codes for primary diagnosis of outpatient clinic visits and hospitalisations.

### Definitions

IHD was defined as ICD-10 codes I20–I25 and ICD‑9 codes 410–414, with the exception of the following ICD-10 and ICD‑9 codes for non-atherosclerotic disease: I201 (coronary spasm), I240 (thrombosis not resulting in myocardial infarction), I241 (Dressler’s syndrome), I254 and 414.10/414.11 (coronary aneurysm or dissection) and I255 (ischaemic cardiomyopathy).

The European LDL‑c target was defined as < 1.4 mmol/l according to the 2019 ESC/EAS Guidelines for the management of dyslipidaemias, [4] and the European SBP target was defined as < 130 mm Hg according to the 2021 ESC Guidelines on CVD prevention in clinical practice [5]. The Dutch LDL‑c and SBP targets were defined as < 2.6 mmol/l and < 140 mm Hg, respectively, according to the 2018 Dutch guidelines on cardiovascular risk management [6].

### Outcome measures

The primary outcome was the potential benefit (expressed as gain in cardiovascular event-free years) of reaching LDL‑c and SBP targets according to Dutch versus European prevention guidelines. Secondary outcomes included the potential decrease of 10-year and lifetime risks of major recurrent CV events and the percentage of patients with LDL‑c and SBP measurements who reached LDL‑c and SBP targets according to Dutch versus European guidelines.

### Statistical analysis

Patient characteristics of the study cohort and of patients excluded due to missing values were compared to investigate potential healthy participant selection bias using *t*-tests for continuous variables, Wilcoxon-rank sum tests for non-continuous variables and Fisher exact tests for categorical variables, with a two-sided *p*-value < 0.005 considered to be statistically significantly different.

To determine the plausibility of genuine missing values for LDL‑c and SBP, instead of being a result of limited data availability, we examined the presence of other laboratory results pertaining to these patients. First, the percentage of patients reaching LDL‑c and SBP targets according to Dutch versus European guidelines was calculated. Second, 10-year and lifetime (i.e. until the age of 90 years) risks of recurrent myocardial infarction, stroke or vascular death were estimated using the Fine and Grey SMART-REACH model—a competing risks prediction model used for secondary prevention—based on the following CVD risk factors: age, sex, last smoking status, diabetes mellitus, number of CVD manifestations (coronary artery disease, cerebrovascular disease and/or peripheral artery disease), atrial fibrillation, heart failure (HF), geographical region (the Netherlands), and lowest systolic blood pressure, total cholesterol level and creatinine level available. Total cholesterol was calculated as follows: lowest available LDL-c + high-density lipoprotein cholesterol +0.2*triglycerides. Third, the effect of reaching the LDL‑c or SBP target was calculated by applying meta-analysis—derived hazard ratios to each individual’s estimated cardiovascular risk [10, 11]. For every 10-mm Hg reduction of the patient’s current SBP, the SMART-REACH model assumes a hazard ratio of 0.80. [10] For every mmol/l reduction of the patient’s current LDL‑c level, a hazard ratio of 0.78 was applied [11]. Estimates of risk and treatment effect were performed for the overall cohort and for subgroups that did not reach Dutch or European targets for both LDL‑c and SBP.

All analyses were stratified by sex. Missing risk factors needed for the SMART-REACH risk estimation were imputed using mean/median imputation, which has been shown to be a robust method for dealing with missing values in CVD prediction models [12]. Numbers of missing predictors, imputation values and a comparison of levels in complete cases versus the imputed cohort can be found in Table S1 in the Electronic Supplementary Material. A sensitivity analysis was performed for complete cases. All statistical analyses were performed using R statistical software (version 4.1.3).

## Results

### Patient population

**Fig. 2:**
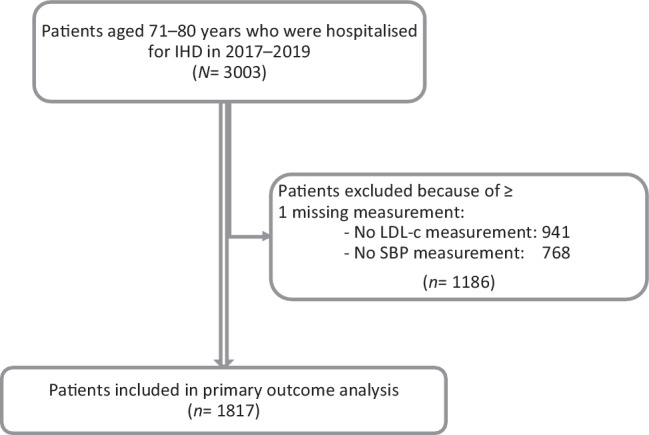
Flowchart of study population. Data on retrospective cohort were extracted from PHARMO Database Network. *IHD* ischaemic heart disease, *LDL‑c* low-density lipoprotein cholesterol, *SBP* systolic blood pressure

Of the 3003 patients hospitalised for IHD from 2017 through 2019, 1186 (39%) had missing LDL‑c and/or SBP measurements, of whom 523 (17% of the overall cohort) missed both values. In total, 1817 patients (36% women) for whom both measurements were available were eligible for primary outcome analysis (Fig. 2). Median age during index hospitalisation was 74 years (interquartile range (IQR): 72–77), and median follow-up duration was 33 months (IQR: 24–41), with 52 patients dying before the end of study period.

Tab. 1 compares characteristics of patients without SBP and/or LDL‑c measurements (*n* = 1186) to those included in the primary outcome analysis. Patients with missing values were more likely to have a first diagnosis of acute coronary syndrome (50% vs 42%) or a history of HF (19% vs 13%), and they had a shorter median follow-up period (25 vs 33 months) due to a higher mortality rate (10% vs 3%). For 96% of the patients with missing LDL‑c and/or SBP values, other laboratory results were available during follow-up. In this group, 245 (21%) had an LDL‑c measurement (mean ± standard deviation (SD): 1.9 ± 0.8 mmol/l) (but no SBP measurement), of whom 80% met the Dutch target and 22% met the European target. For SBP, 418 (35%) had a measurement (mean ± SD: 129 ± 21 mm Hg) (but no LDL‑c measurement), of whom 69% met Dutch and 52% met European targets.

**Table 1:** Patient characteristics

Characteristic	Eligible patients (*N* = 3003)	Patients included in primary analysis (*n* = 1817)	Patients without LDL‑c and/or SBP measurements (*n* = 1186)	*P*-value^a^
*Socio-demographics*				
Age at event, years	74 (72–77)	74 (72–77)	74 (72–77)	
Women	36	36	32	
Urban	45	45	46	
*Index hospitalisation*				
Diagnosis of acute coronary syndrome	42	42	50	< 0.001
Deceased before end of study period	3	3	10	< 0.001
Follow-up time, months	33 (24–41)	33 (24–41)	25 (17–36)	< 0.001
*Cardiovascular medical comorbidity*				
Prior ischaemic heart disease	48	48	41	< 0.001
Cerebrovascular disease	9	9	8	
Peripheral arterial disease	6	6	7	
Diabetes mellitus	15	15	15	
Heart failure	13	13	19	< 0.001
Atrial fibrillation	19	19	21	
*Reaching lifestyle targets*^*b*^				
Non-smoking	91	91	86	
BMI, kg/m^2^ mean ± SD	27 ± 4	27 ± 4	27 ± 5	
BMI < 25 kg/m^2^	33	33	34	
BMI < 30 kg/m^2^	79	79	77	

Data are median (interquartile range), %, or mean ± standard deviation

^a^ Smoking status and body mass index (*BMI*) based on last status recorded by general practitioner

^b^ Comparison between patients with and without low-density lipoprotein cholesterol (*LDL‑c*) and/or systolic blood pressure (*SBP*) measurements. *P*-value only provided if considered significant (*p* < 0.05)

### Current risk factor levels

**Fig. 3:**
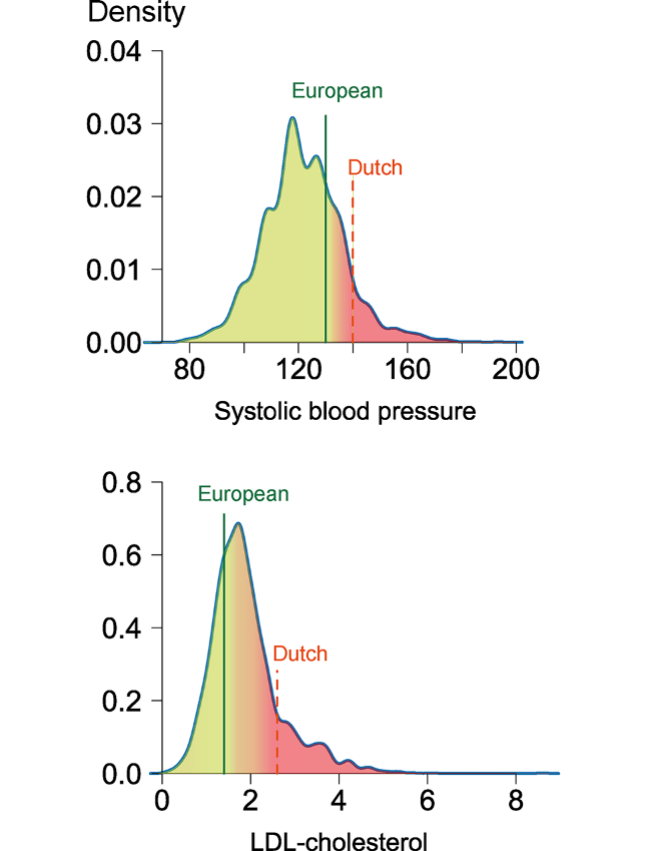
Distribution of systolic blood pressure (*SBP*) and low-density lipoprotein cholesterol (*LDL‑c*) levels. Density plots of lowest SBP or LDL‑c levels available from each individual after index hospitalisation. *Vertical lines* show European (*solid*) and Dutch (*dashed*) guideline-recommended targets

Distributions of LDL‑c and SBP levels are shown in Fig. 3. Mean ± SD SBP was 125 ± 16 mm Hg, and median LDL‑c level was 1.8 mmol/l (IQR: 1.4–2.2). Dutch targets for LDL‑c and SBP were both reached by 84%, whereas European targets were reached by 23% and 61%, respectively (Tab. 2). With these risk factor levels, median 10-year and lifetime risks for recurrent events were 29% (IQR: 24–36%) and 39% (IQR: 35–46%), respectively, for the overall population.

**Table 2:** Potential benefit of reaching guideline-recommended targets for SBP and LDL‑c

Target	Patients (*n* = 1817)		Men (*n* = 1165)		Women (*n* = 652)		*P*-value^a^
**Reaching guideline-recommended targets**							
*Dutch*							
– SBP < 150 mm Hg^b^	94		94		94		
– SBP < 140 mm Hg	84		85		83		
– LDL-c < 2.6 mmol/l	84		88		77		< 0.01
*European*							
– SBP < 130 mm Hg	61		62		60		
– LDL-c < 1.8 mmol/l^b^	49		53		41		< 0.01
– LDL-c < 1.4 mmol/l	23		25		19		0.02
**Overall SMART-REACH estimates**							
*Current*							
– 10-year risk of recurrent events	29 (24–36)		30 (26–38)		26 (22–32)		< 0.01
– Lifetime risk of recurrent events	39 (35–46)		41 (36–47)		37 (33–43)		< 0.01
– Event-free life expectancy	84 (82–86)		83 (82–84)		86 (84–87)		< 0.01
*If Dutch targets are met by all patients*							
– 10-year risk of recurrent events	28 (23–35)		29 (24–37)		25 (21–31)		< 0.01
– Lifetime risk of recurrent events	38 (33–44)		39 (34–36)		25 (30–42)		< 0.01
– Benefit in event-free years	0.0 (0.0–0.1)		0.0 (0.0–0.0)		0.0 (0.0–0.4)		< 0.01
*If European targets are met by all patients*							
– 10-year risk of recurrent events	24 (19–32)		26 (21–34)		21 (16–27)		< 0.01
– Lifetime risk of recurrent events	34 (27–41)		36 (29–43)		31 (24–38)		< 0.01
– Benefit in event-free years	0.5 (0.2–1.2)		0.4 (0.1–0.9)		0.7 (0.3–1.7)		< 0.01
**Subgroup reaching Dutch but not European SBP or LDL‑c targets**							
***N***	**1281**		**833**		**448**		
Age, years	74 (72–77)		74 (72–77)		74 (72–77)		
SBP ≥ 130 mm Hg	46		42		48		
LDL-c ≥ 1.4 mmol/l	93		93		94		
*SMART-REACH estimates*							
– Current 10-year risk of recurrent events	28 (24–35)		30 (35–37)		25 (22–31)		< 0.01
– Current lifetime risk of recurrent events	39 (34–45)		40 (35–46)		37 (33–43)		< 0.01
– Benefit in event-free years if European targets are met	0.6 (0.3–1.0)		0.5 (0.2–0.8)		0.8 (0.4–1.4)		< 0.01
**Subgroup not reaching Dutch SBP or LDL‑c targets**							
***N***	** 501**		**272**		**229**		
Age, years	75 (72–78)		75 (72–78)		75 (72–78)		
SBP ≥ 140 mm Hg	56		63		48		< 0.01
LDL-c ≥ 2.6 mmol/l	57		50		66		< 0.01
*SMART-REACH estimates*							
– Current 10-year risk of recurrent events	28 (23–34)		30 (25–37)		25 (21–30)		< 0.01
– Current lifetime risk of recurrent events	38 (33–44)		39 (35–45)		35 (31–41)		< 0.01
– Benefit in event-free years if Dutch targets are met	0.6 (0.2–1.2)		0.5 (0.2–1.0)		0.7 (0.3–1.5)		< 0.01
– Benefit in event-free years if European targets are met	1.7 (1.2–2.5)		1.5 (1.0–2.1)		2.0 (1.5–2.9)		< 0.01

Data are % or median (interquartile range)

^a^ *P*-value indicates difference between men and women and is only provided if considered significant (*p* < 0.05)

^b^ Minimum targets were < 150 mm Hg for systolic blood pressure (*SBP*) and < 1.8 mmol/l for low density lipoprotein cholesterol (*LDL‑c*), as per Dutch and European guidelines. These targets were applied to all patients, including frail elderly and those intolerant of more intensive medication. Optimal targets were < 140 mm Hg (Dutch) and < 1.4 mmol/l (European), respectively

### Potential benefit of guideline-based targets

**Fig. 4:**
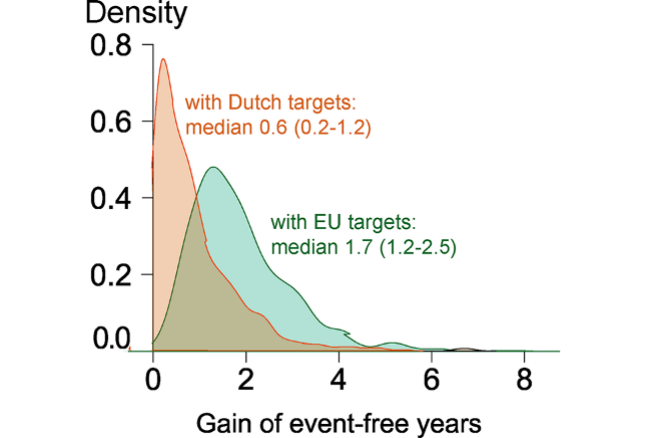
Gain of cardiovascular event-free years that could be reached with Dutch versus European (*EU*) treatment targets for systolic blood pressure and low-density lipoprotein cholesterol in subgroup that currently did not reach both Dutch targets (*n* = 501)

On top of current risk levels, reaching Dutch or European targets would result in a gain of median 0.0 (IQR: 0.0–0.1) and 0.5 (IQR: 0.2–1.2) event-free years, respectively. In 1281 patients reaching Dutch but not European targets, the additional effect of reaching European targets was 0.6 years (IQR: 0.3–1.0). The greatest effect could be reached in patients who currently did not meet Dutch targets for both LDL‑c and SBP (*n* = 501), i.e. a gain of median 0.6 (IQR: 0.2–1.2) and 1.7 (IQR: 1.2–2.5) event-free years by reaching Dutch versus European targets, respectively (Fig. 4).

Sensitivity analysis of complete cases (*n* = 1511) showed similar results (see Table S1 in Electronic Supplementary Material).

### Sex differences

Mean SBP levels and targets were comparable for both sexes. However, LDL‑c levels were higher in women compared with men (median (IQR): 1.9 (1.5–2.4) vs 1.7 (1.4–2.1); *p* < 0.01), and therefore fewer women reached Dutch (77% vs 88%; *p* < 0.01) and European (19% vs 25%; *p* = 0.02) LDL‑c targets (Tab. 2). Furthermore, women had a longer event-free life expectancy (median (IQR): 86 (84–87) vs 83 (82–84) years; *p* < 0.01). Consequently, the potential lifetime benefit of (strict) risk factor management was estimated to be greater in women than men (*p* < 0.01 for all comparisons) (Tab. 2).

## Discussion

In a contemporary, real-world Dutch population cohort, the majority of patients > 70 years of age with IHD met Dutch guideline-recommended targets for both LDL‑c and SBP. Conversely, European targets were met by 23% (LDL-c) and 61% (SBP). Adhering to European targets resulted in a greater gain of event-free years compared with Dutch targets. These benefits were greater in patients with poorer risk factor control. As risk factor control was worse in women compared with men, the potential lifetime benefit in women was estimated to be greater. Our findings suggested that adopting European targets for all patients > 70 years yields the greatest benefit in terms of event-free years, especially in those with poorer risk factor control, provided the regimen is well-tolerated.

In our study population of 1817 patients with documented LDL‑c and SBP measurements, 84% reached the Dutch guideline targets. However, this should be interpreted against the background that 39% of the total cohort of 3003 patients had missing LDL‑c and/or SBP measurements in our database. The likelihood that these measurements are true missing values (i.e. measurement not performed instead of missing in our data collection) is considerable, as we had access to laboratory data for all these patients and 96% of them had other laboratory results during follow-up, such as creatinine levels. The large number of missing measurements could be due to the fact that patients had achieved the (Dutch) targets prior to hospital admission, were already at maximally tolerated therapy or had a poor prognosis. However, the large percentage of missing measurements in our study is alarming and warrants further investigation.

Currently, insufficient risk factor control is often a result of clinical inertia, which includes patient, physician and system factors [13]. The stricter treatment targets outlined in the European guidelines are supported by evidence, demonstrating these goals are safe for many older patients and that they are associated with significantly lower risks of major stroke, HF and CV death [4, 7, 8]. Consequently, when discussing intensification or reduction of risk factor treatment for individuals older than 70 years, it is imperative to consider life expectancy, not solely age, in addition to safety and tolerability.

The latest European preventive guidelines introduce a differentiation between 10-year risk and lifetime projected risk and benefits. This distinction aims to facilitate discussions about treatment options and consideration of long-term health benefits, which are greater in younger compared with older patients. In general, a 1-year gain in cardiovascular event-free life expectancy is considered clinically meaningful; however, this may vary individually, and older patients tend to prefer greater gains than younger patients [14]. In our study, we observed median estimated lifetime benefits up to 1.7 years, indicating that individuals aged ≥ 70 years can derive substantial benefits from stricter risk factor targets.

### Strengths and limitations

There are several strengths to our study. First, we analysed a large, real-world database of healthcare settings, which included general practices, clinical laboratories and hospitals [9]. The data linkage in the PHARMO Database Network provides a unique opportunity to fill gaps in patient journeys and investigate real-world treatment levels, which cannot be addressed in most single (unlinked) databases. Second, as the PHARMO Database Network follows patients regardless of age and sex or other in- or exclusion criteria, our study had minimal selection bias. Finally, we calculated risk estimations using the innovative and validated SMART-REACH model, which supports treatment (goal) decisions with outcomes that are relevant to clinical practice.

Some aspects of our study deserve consideration. First, our primary outcome was not *observed* mortality and morbidity but contained an estimate, with inherent uncertainty thereof. Second, we were limited to including patients from the PHARMO Database Network for whom all required data linkages were accessible, thus restricting our sample size. Furthermore, we had to exclude patients without LDL‑c and SBP measurements from our primary outcome analysis, which may have introduced healthy participant selection bias. However, we have provided insight into this in Tab. 1. Finally, we did not include information on the type of medication prescribed and dispensed, which could have provided insight into treatment effects and unavoidable or justifiable causes of unmet treatment targets.

## Conclusion

In a contemporary, real-world Dutch population cohort aged 71–80 years with IHD, LDL‑c and/or SBP measurements were missing for a considerable proportion of patients. This implied considerable room for improved rates of risk quantification and reduction and warrants further investigation. Among patients with both LDL‑c and SBP measurements, the majority reached Dutch targets but not European targets. The greatest benefit could be achieved by implementing European targets, especially in patients with poorer risk factor control.

### Supplementary Information

Table S1 SMART-REACH predictor values before and after imputation, Table S2. Comparison of results in complete cases vs. (imputed) study cohort
