# Supplementary material for: Impact of implementing Dutch versus European guideline risk factor targets in older patients with ischaemic heart disease
Source: Neth Heart J. 2023 Oct 23;32(1):45–54. doi: 10.1007/s12471-023-01823-x (PMC10781920; doi:10.1007/s12471-023-01823-x)
Supplement: Supplementary file 1 — Table S1 SMART-REACH predictor values before and after imputation, Table S2. Comparison of results in complete cases vs. (imputed) study cohort [file 12471_2023_1823_MOESM1_ESM.docx]

|  | **Missings after hospitalisation**  n (%) from 1,817 | **Sensitivity analysis Complete cases**  N= 1,511 | **Main analysis: imputation**  N=1,817 |
| --- | --- | --- | --- |
| **SMART-REACH predictors for risk** |  |  |  |
| Age, median (IQR) | 0 (0) | 74 (72-77) | 74 (72-77) |
| Female sex | 0 (0) | 36% | 36% |
| Diabetes mellitus | 0 (0) | 16% | 15% |
| Coronary artery disease | 0 (0) | 100% | 100% |
| Cerebrovascular disease | 0 (0) | 10% | 9% |
| Peripheral artery disease | 0 (0) | 7% | 6% |
| Atrial fibrillation | 0 (0) | 19% | 19% |
| Heart failure | 0 (0) | 13% | 14% |
| Current smoking | 276 (15) | 8.4% | 8.5% |
| Systolic blood pressure (mmHg), median (IQR) | 0 (0) | 123 | 124 |
| Total cholesterol (mmol/L), median (IQR) | 14 (0.8) | 3.3 | 3.3 |
| Creatinin (umol/L or mg/dL), median (IQR) | 48 (2.6) | 81 | 82 |
| Residence | 0 (0) | Netherlands | Netherlands |

**Table S1** SMART-REACH predictor values before and after imputation

Values are shown as percentage if categorical variable (1=yes, 0=no), median (IQR) if continuous variable unless otherwise specified. Sensitivity analysis: SMART-REACH predictions in complete cases (N=1511). Main analysis: in 75 patients a historical smoking status before hospitalisation was available: if last smoking status before hospitalisation was ‘never’ or ‘former’, this last observation was carried forward. If last smoking status was ‘current’, smoking status were replaced by 0.5, as 50% of smokers continues smoking after an coronary hospitalisation.^1^ If there was no historical smoking status, missing values were replaced by the mean value of the cohort (with 8.9% smoking, all missing smoking status were replaced by the value 0.089). Missing continuous variables (total cholesterol and creatinine) were replaced by the median value of the cohort.

1. Snaterse M, Deckers JW, Lenzen MJ, Jorstad HT, De Bacquer D, Peters RJG*, et al.* Smoking cessation in European patients with coronary heart disease. Results from the EUROASPIRE IV survey: A registry from the European Society of Cardiology. *Int J Cardiol* 2018;**258**:1-6. doi: 10.1016/j.ijcard.2018.01.064

|  | **Sensitivity analysis Complete cases (N=1,511)** | **Main analysis: Mean/median imputation (N=1,817)** |
| --- | --- | --- |
| **Reaching guideline-dependent targets, n (%)** |  |  |
| **Dutch** |  |  |
| SBP<150 mmHg | 96% | 94% |
| SBP<140 mmHg | 87% | 84% |
| LDL-C<2.6 mmol/L | 86% | 84% |
| **European** |  |  |
| SBP<130 mmHg | 64% | 61% |
| LDL-C<1.8 mmol/L | 49% | 49% |
| LDL-C<1.4 mmol/L | 24% | 23% |
|  |  |  |
| **SMART-REACH estimates, median (IQR)** |  |  |
| **Current** |  |  |
| Ten year risk of recurrent events | 29% (24-36) | 29% (24-36) |
| Lifetime risk of recurrent events | 39% (35-46) | 39% (35-46) |
| **If Dutch targets are met** |  |  |
| Ten year risk of recurrent events | 28% (23-35) | 28% (23-35) |
| Lifetime risk of recurrent events | 39% (34-45) | 38% (33-44) |
| Benefit in event-free years | 0.0 (0.0-0.0) | 0.0 (0.0-0.1) |
| **If European targets are met** |  |  |
| Ten year risk of recurrent events | 25% (20-32) | 24% (19-32) |
| Lifetime risk of recurrent events | 34% (28-41) | 34% (27-41) |
| Benefit in event-free years | 0.5 (0.1-1.0) | 0.5 (0.2-1.2) |

**Table S2. Comparison of results in complete cases vs. (imputed) study cohort**
